# Robotic exoskeleton embodiment in post-stroke hemiparetic patients: an experimental study about the integration of the assistance provided by the REFLEX knee exoskeleton

**DOI:** 10.1038/s41598-023-50387-8

**Published:** 2023-12-21

**Authors:** Julio Salvador Lora-Millan, Francisco José Sanchez-Cuesta, Juan Pablo Romero, Juan C. Moreno, Eduardo Rocon

**Affiliations:** 1 Electronic Tecnology Area, Rey Juan Carlos University, Madrid, Spain; 2 Facultad de Ciencias Experimentales, Universidad Francisco de Vitoria, Pozuelo de Alarcón, Madrid, Spain; 3 Brain Injury and Movement Disorders Neurorehabilitation Group (GINDAT), Institute of Life Sciences, Francisco de Vitoria University, Pozuelo de Alarcón, Madrid, Spain; 4 Brain Damage Unit, Hospital Beata María Ana, Madrid, Spain; 5 Neural Rehabilitation Group, Cajal Institute, Spanish National Research Council (CSIC), Madrid, Spain; 6 Centro de Automática y Robótica, Spanish National Research Council (CSIC), Madrid, Spain

**Keywords:** Biomedical engineering, Motor control

## Abstract

Hemiparetic gait is the most common motor-disorder after stroke and, in spite of rehabilitation efforts, it is persistent in 50% of community dwelling stroke-survivors. Robotic exoskeletons have been proposed as assistive devices to support impaired joints. An example of these devices is the REFLEX knee exoskeleton, which assists the gait of hemiparetic subjects and whose action seems to be properly embodied by stroke survivors, who were able to adapt the motion of their non-assisted limbs and, therefore, reduce their compensation mechanisms. This paper presents an experimental validation carried out to deepen into the effects of REFLEX’s assistance in hemiparetic subjects. Special attention was paid to the effect produced in the muscular activity as a metric to evaluate the embodiment of this technology. Significant differences were obtained at the subject level due to the assistance; however, the high dispersion of the measured outcomes avoided extracting global effects at the group level. These results highlight the need of individually tailoring the action of the robot to the individual needs of each patient to maximize the beneficial outcomes. Extra research effort should be done to elucidate the neural mechanisms involved in the embodiment of external devices by stroke survivors.

## Introduction

Stroke is one of the most common causes of long-term disability worldwide^1^, and it is estimated that the number of people living with stroke will increase by 27% between 2017 and 2047 in the European Union (EU)^2^. 65% of people who suffered a stroke show gait impairments that difficult the performance of daily life activities^3^ and reduce their autonomy and quality of life ^4^, being the hemiparetic gait the most common post-stroke gait disturbance^5^. Hemiparetic gait is characterized by a strong asymmetric pattern due to contralateral motor weakness, motor control deficits, sensory and/or proprioceptive loss, and/or ataxia^5^. It might cause several consequences such as musculoskeletal pathologies in the non-paretic limb due to the development of compensation strategies to deal with gait impairments^6^, falls due to instability, slow gait velocity, or increased energy consumption^7,8^.

Motor recovery after stroke remains a clinical challenge^9^ since asymmetric gait can be resistant to intervention^10^ and it is still present in 50% of community-dwelling chronic stroke survivors^7^. In this context, robotic exoskeletons have been presented as alternative devices to assist the gait of post-stroke hemiparetic subjects^11–16^. The full exploitation of this technology will be achieved when these devices are embodied by users, i.e. the wearer considers that the device's action is performed by their own body rather than by an external tool^17–19^.

Following this approach, we have developed the REFLEX exoskeleton to assist the paretic knee function of hemiparetic walkers^20^. Its preliminary validation was promising and suggested the proper embodiment of the REFLEX’s action in a small cohort of three stroke patients. Results showed the decrease of compensatory mechanisms developed by the non-paretic leg while assisting exclusively the paretic limb^20^. The achieved technology embodiment would imply that the compensation mechanisms would no longer be necessary since the joints are assisted by the action of the robot. However, some aspects of this gait adaptation still remain elusive.

In this paper, we aim at evaluating the proper neuromuscular integration of REFLEX's action which will lead to adaptations and gait changes. These reported changes can be kinematic adaptations, as preliminary results seemed to indicate^20^, but also should be accompanied by muscular adaptations that indicate a change in the neural gait control. Recently, authors described muscular adaptations due to robotic assistance. Gordon et al.^21^ reported that unimpaired subjects could reduce soleus activity while assisted by an ankle exoskeleton. Similarly, the ankle exoskeleton used by Steele et al.^22^ also reduced healthy ankle plantarflexor activity due to its assistance during gait. Wehbi et al.^23^ used a knee exoskeleton to assist healthy knee flexion during swing, reducing the muscular effort of the short head of the biceps femoris and the vastus medialis. Lee et al.^24^ also reduced healthy knee extensor activity using a robotic knee exoskeleton, although they assisted the stance phase of the gait and validated the system during inclined and declined walking. Instead of a rigid robot, Sridar et al.^13,25^ used a soft knee exoskeleton to assist knee extension during gait, reducing quadriceps activity in healthy subjects. Acosta-Sojo et al.^26^ also evaluated the muscular response of healthy subjects to the assistance provided by an ankle exoskeleton during walking. Sixty percent of participants reduced medial gastrocnemius activity and 80% reduced tibialis anterior activity due to the robot assistance, authors pointed out that this reduction may affect the antagonist muscular effort as a result. However, muscular responses highly varied between individuals, hampering the identification of global effects in this sense. Due to this discrepancy, their study concluded the necessity of understanding these individual adaptations to tailor the exoskeleton performance.

The above-mentioned papers recruited unimpaired walking subjects to validate the presented devices. Their results suggested that healthy subjects are able to embody the robotic assistance, since they reduce muscular activity and, therefore, the energetic cost of walking, increasing their endurance. Most published research works about robotic assistance in hemiparetic patients after stroke are focused on achieving an autonomous and stable gait^27^. Although some authors have reported muscular adaptations, these can not be considered as signs of the device’s embodiment. For example, Tan et al.^28^ reported muscular adaptations in eight stroke survivors, but after a rehabilitation therapy instead of being adaptations during the use of the robot. Androwis et al.^29^ also reported muscular adaptations in five stroke survivors, but they wore a complete robotic exoskeleton intended to rehabilitate rather than assist the user. In spite of these results, there is still a lack of understanding of how impaired walking subjects react to robot assistance, especially whether they were able to integrate the robot action in the neural gait control and consequently adapt their muscular activity to it.

The aim of this paper is to further understand the effects of the REFLEX assistance in hemiparetic patients, focusing both on the kinematic adaptations and the muscular response that would indicate the embodiment of the robotic action^30^. Concretely, the developed device aims at improving the gait symmetry of the patients while wearing the robot and reducing the compensation mechanisms developed by the sound leg (including the excessive involvement of the healthy limb) because of the embodiment of the robotic exoskeleton.

Firstly, this paper slightly describes the exoskeleton and the experimental protocol developed to evaluate above-mentioned effects, then the metrics and the conducted data analyses are presented. The obtained results are reported and analysed individually per subject and globally to verify our hypothesis regarding the embodiment of the REFLEX exoskeleton. The obtained conclusions are discussed at the end of this work.

## Methods

### Experimental procedure

Post-stroke hemiparetic participants were recruited for this study. Both, males and females are considered and between 18 and 85 years old. These patients had to show hemiplegic gait after a ischemic or haemorrhagic stroke. Several exclusion criteria were considered: acute musculoskeletal, cardiopulmonary or neurological diseases, excessive spasticity or mobility restriction in any joint of the lower limb, pain due to impaired mobility and inability to understand and report simple information.

A total of seven hemiparetic stroke patients were recruited for this study (demographic data is summarized in supplementary table 1 of the Supplementary Material). The study was conducted according to the Declaration of Helsinki after being approved by the local ethics committee. Written (signed) informed consent was obtained from all enrolees. Trial and protocol were publicly registered in ClinicalTrials on 17/11/2021; Trial id: NCT05138211.

**Figure 1:**
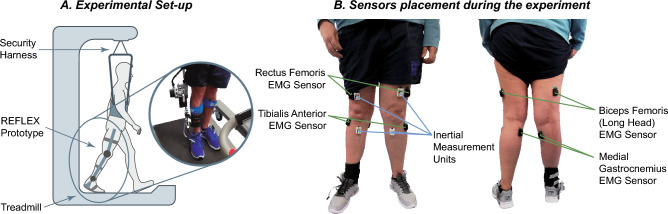
Panel (**A**): Experimental set-up during the session. A subject was wearing the REFLEX prototype while walking on the treadmill. He/she was also wearing a security harness that did not support the patient's weight. Panel (**B**): Sensors set-up during the experimental validation. Anterior (left figure) and posterior (right figure) view of the sensors set-up during the experimental validation. EMG sensors were placed in the Rectus Femoris, Tibialis Anterior, Biceps Femoris (long head), and Medial Gastrocnemius. Inertial sensors were placed in both thighs and shanks of the subject.

Participants were instructed to wear the REFLEX prototype^20^ in the paretic leg while walking on a treadmill. As a safety measure, participants were connected to a security harness that did not support any of the patient's weight. Panel A of Fig. 1 shows one of the patients during the experimental sessions.

The presented protocol aimed at assessing the response of the participants to the provided assistance. Specifically, the effects on their gait and the possible embodiment of this assistance that should be reflected by changes in muscular activity. Additionally, this protocol also aimed to assess participants’ gait kinematics and muscular activity under different gait speeds, to elucidate if walking velocity affects the embodiment of the exoskeleton.

For this experimental set-up, wireless EMG sensors (Trigno Avanti sensors from Delsys Inc, USA) were used to record muscular activity from representative lower limb muscles of both legs: rectus femoris, biceps femoris (long head), tibialis anterior and medial gastrocnemius. The *Seniam* guidelines^31^ were followed to ensure the proper quality of the EMG recordings. In this regard, the muscles’ bellies were identified to place the electrodes after shaving and cleaning the zone with alcohol. Additionally, inertial sensors were placed in both shanks and thighs to acquire the kinematics of both hips and knees. Panel B of Fig. 1 shows the referred sensor set-up.

**Figure 2:**
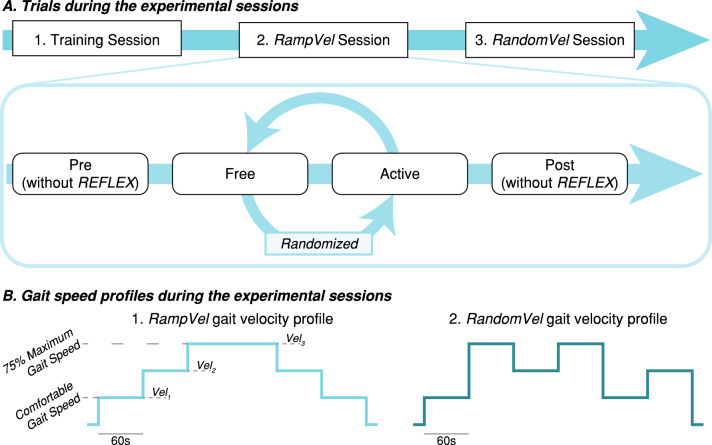
Description of the experimental protocol. Panel (**A**) shows the trials that composed each of the two experimental sessions. Panel (**B**) shows the gait speed profile used during both sessions. The gait speed varied on a patient-dependent scale from a comfortable level to 75% of the maximum tolerable speed in epochs of 60 s. During the *RampVel* session, the gait speed varied from the minimum to the maximum value and decreased afterward. Conversely, during the RandomVel session, the gait speed randomly varied using the same values as in the previous session.

The experimental protocol was composed of one training session and two measurement sessions separated by one or two days each, as illustrated in Fig. 2, panel A. The objectives of the training session are: (1) to determine the assistance level provided by the exoskeleton according to the clinical criteria, (2) to establish the comfortable and maximum gait speed of the patient while walking with and without the robot, and (3) to enable patients to familiarize to the use and assistance provided by REFLEX. All involved patients were naïve to the use of the robot, being the first time that they use it. During this training session, patients walked with the device for approximately thirty minutes, with three resting periods of five minutes.

Also during this training session, several clinical tests were carried out to measure the physical and cognitive state of the patients and their walking ability. Besides, a sensory examination was carried out as a safety procedure to evaluate the participant’s perception and ability to detect forces, pressure, and light touch. The purpose of this procedure was to ensure whether we could rely on the patients’ perception to avoid excessive pressure that could be harmful. All patients showed limited pressure and touch perception in their paretic legs.

The measurement sessions were divided into four different trials (see Fig. 2, panel A). During the first and last trials, namely *Pre* and *Post* trials, the patients did not wear the robotic exoskeleton, as they were intended to measure their basal state and potential immediate effects after using the robot. The second and third trials were randomly assigned to either allow unrestricted motion by mechanically decoupling the REFLEX’s joint (*Free* trial) or to provide gait assistance (*Active* trial). Resting periods of ten minutes at least were interleaved between trials.

REFLEX was controlled with two strategies: one controller replicated the kinematic trajectory of the non-assisted limb (named *Echo* control), the other controller used a normal (healthy) gait patter as reference trajectory (named *Pattern* control). During our preliminary validation^20^ results showed that assisting hemiparetic patients through the *Pattern* strategy led to a greater room for improvements and therefore achieved better outcomes. Due to this, in this protocol only the *Pattern* strategy was used.

Two different measurement sessions were proposed, namely *RampVel* and *RandomVel*, depending on the order of the commanded gait speed. These two sessions have the purpose of evaluate if the order of the gait speed changes have any effect in the results. During the training session, the gait speed levels of each patient were determined. Gait speed varied from the identified comfortable gait speed $($) to the 75% of their maximum gait speed (${\text{vel}}_{{3}}$), with an equidistant intermediate speed level (${\text{vel}}_{{2}}$). Each trial involved two repetitions of each velocity, lasting 60 s each, so a total of four epochs were performed at the same speed. In *RampVel* trials, gait velocity increased from the slowest to the fastest and returned to the slowest afterward. Conversely, the order of each velocity was randomly set in *RandomVel* trials. Panel B of Fig. 2 represents an example of the gait speed profile for a *RampVel* and a RandomVel trial. In these sessions, baseline EMG signals were recorded to determine the noise level of the sensors previously to carry out the trials.

### Metrics and data analysis

**Figure 3:**
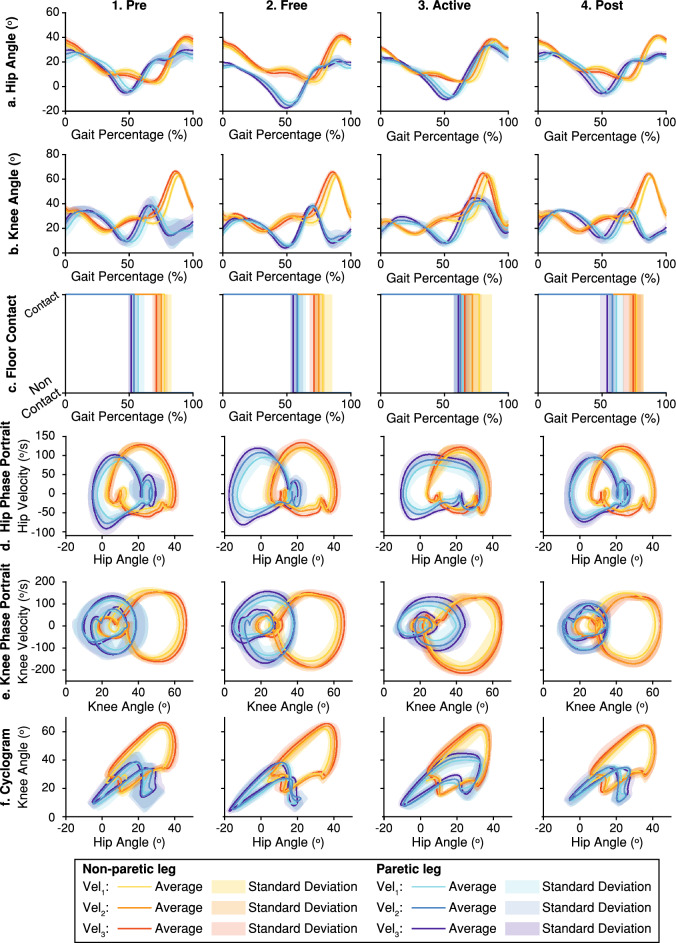
Example of step average kinematic data for one patient. Columns 1–4 represent the results obtained during each trial (*Pre*, *Free*, *Active* and *Post* trials respectively). Each row shows different kinematic information: hip and knee flexion angle (rows **a** and **b**), contact of the foot with the floor (row **c**), hip and knee phase portraits (rows **d** and **e**), and the hip-knee cyclogram (row **f**). The information of each leg and gait velocity is represented using a different color; solid lines represent the average data while semi-transparent areas represent the dispersion of the data (see the legend for details).

Data recorded during the experiment was segmented between consecutive heel strikes to divide it into steps and classified according to the commanded gait speed. Step data were normalized from 0 to 100% of the gait cycle. Figure 3 shows an example of average step data for one patient.

A threefold analysis was conducted with the recorded information to assess the gait adaptation to the provided assistance and the embodiment of this technology. Concretely, gait features symmetry, motion kinematics, and muscular activity were evaluated. The datasets used and/or analysed during the current study are available from the corresponding author on reasonable request.

### Assessment of gait features symmetry

Four gait features were selected for this analysis: step length, step time, step velocity and stance/swing ratio (defined as the ratio between stance time $\left( {T_{stance} } \right)$ and swing time $\left( {T_{swing} } \right)$, Eq. (1)

1 $${\raise0.7ex\hbox{${St}$} \!\mathord{\left/ {\vphantom {{St} {Sw}}}\right.\kern-0pt} \!\lower0.7ex\hbox{${Sw}$}}\left( \% \right) = \frac{{T_{stance} }}{{T_{swing} }}\cdot100$$

**Figure 4:**
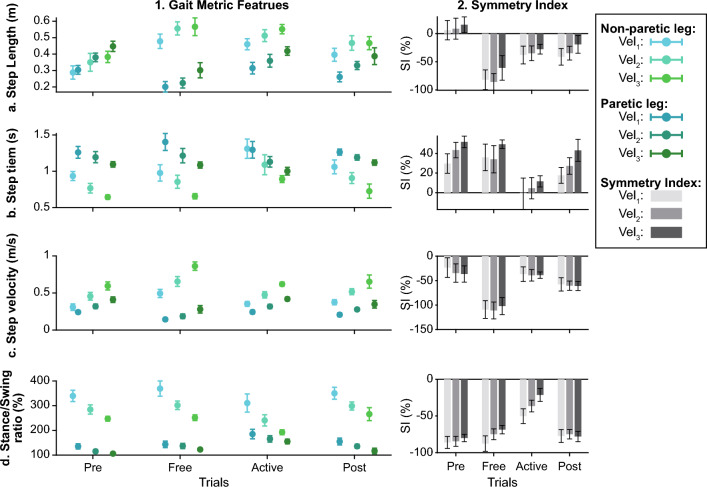
Example of gait feature metrics for one patient. Rows a-d show the information for different gait features: step length, step time, step velocity, and stance/swing ratio, respectively. Column 1 represents the metric distribution (mean +/− standard deviation) for each leg and gait speed across trials (see the legend for details). Column 2 represents the symmetry index of each gait metric across trials and gait speeds.

The symmetry of these metrics was assessed using the Symmetry Index introduced by Arazpour et al.^32^ (Eq. 2). Figure 4 represents the distribution of these metrics for one example subject and their symmetry indexes.

2 $$SI\left( \% \right) = \frac{{\overline{X}_{A} - \overline{X}_{S} }}{{\frac{1}{2}\left( {\overline{X}_{A} + \overline{X}_{S} } \right)}}\cdot100$$

Where $\overline{X}_{A}$ and $\overline{X}_{B}$ are the mean value for a metric in the assisted and sound (or non-assisted) leg, respectively. A SI of zero value means a complete symmetry, and a higher SI means a higher asymmetry in the metric.

### Assessment of motion kinematics

This analysis is based on the hip and knee phase portraits and the hip-knee cyclograms. Phase portraits are representations that show the angular position on the X-axis and the angular velocity on the Y-axis, so the resulting portrait’s shape is representative of the motion's dynamics^20,33^. Cyclograms show the hip flexion angle on the X-axis against the knee flexion angle on the Y-axis. The similarity between two motions was assessed using the similarity index defined in the Eq. (3).

3 $$Sim\left( \% \right) = \frac{A \cap B}{{A \cup B}} \cdot 100$$

where $A$ and $B$ are the areas of two closed shapes (phase portraits or cyclograms), so $A \cap B$ is the common area between them, and $A \cup B$ is the union of both of them.

**Figure 5:**
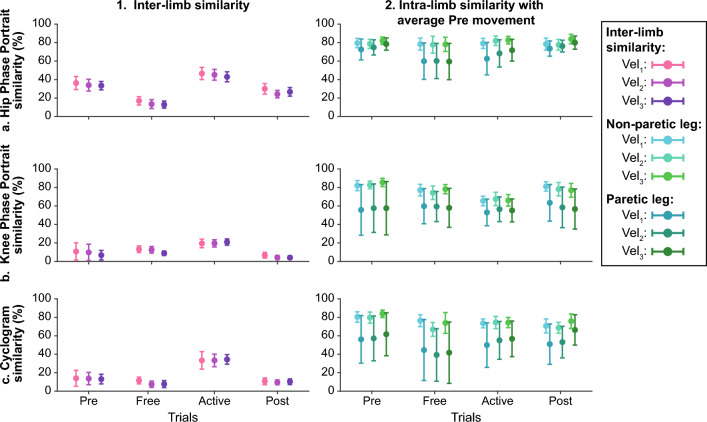
Example of inter-limb and intra-limb motion similarity in one patient. Columns 1 and 2 show the inter-limb motion similarity and the intra-limb motion similarity with the average motion during *Pre* trials. Data distributions are represented through the mean +/− the standard deviation. Rows (**a**–**c**) show the hip phase portrait, knee phase portrait, and cyclogram similarity, respectively. All the information is represented across trials and gait velocity (see the legend for more details).

Figure 5 represents the results of comparisons between limbs motion for one patient. Two aspects were assessed: inter-limb and intra-limb similarity. Inter-limb similarity refers to the comparison between both legs within the same trial. In contrast, intra-limb similarity refers to the comparison between the motion of one leg during any trial and the same leg during the *Pre* trial. In this way, it is possible to find changes in the motion symmetry and identify the origin of these changes. For example, in Fig. 5, the knee phase-portrait shows an increase in inter-limb motion similarity, being these changes due to the evolution of both legs' motion since the comparison between *Pre* and *Active* trials showed differences in both legs.

### Assessment of muscular activity

**Figure 6:**
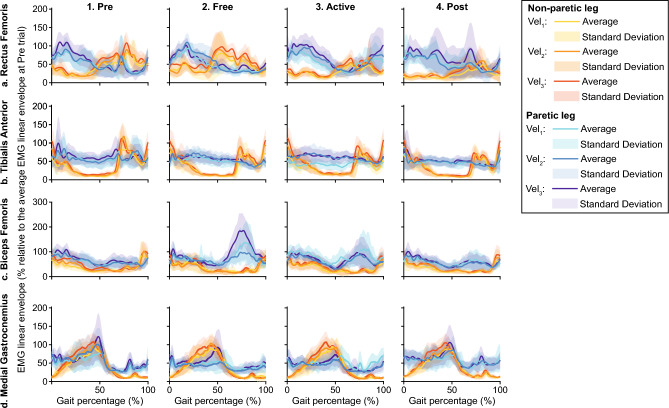
Example of average EMG linear envelopes for one patient. Columns 1–4 represent the results obtained during each trial (*Pre*, *Free*, *Active* and *Post* trials respectively). Each row shows the activation of a representative muscle: Rectus Femoris, Tibialis Anterior, Biceps Femoris, and Medial Gastrocnemius for rows (**a**–**d**). The information of each leg and gait velocity is represented using a different color; solid lines represent the average data while semi-transparent areas represent the dispersion of the data (see the legend for details).

Two aspects were analyzed in the patients' muscular activity: amplitude and timing. The magnitude of this signal was assessed using its linear envelope ($leEMG$). In order to calculate it, Notch filters were used to remove electromagnetic environmental noise at 50 Hz and harmonics; afterward, EMG signals were high-pass filtered (zero-lag 4th order Butterworth, cut-off 20 Hz), rectified, and low-pass filtered (zero-lag 4th order Butterworth, cut-off 10 Hz). Figure 6 represents an example of the average EMG linear envelope for one patient during the gait cycle. Two patients’ Biceps Femoris EMG data had to be removed due to erroneous recordings and improper interactions between the electrodes and the robotic exoskeleton.

**Figure 7:**
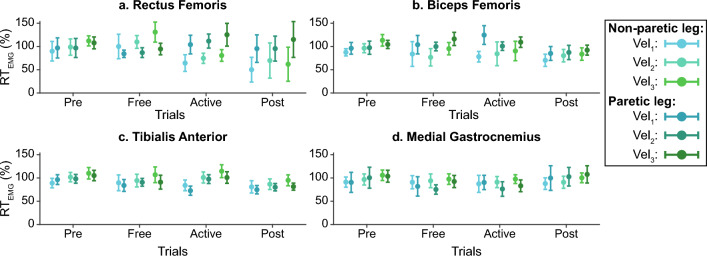
Example of muscular activity amplitude for one patient. Data distributions are represented through the mean +/− the standard deviation. Each panel represents information for a representative muscle: Rectus Femoris, Tibialis Anterior, Biceps Femoris, and Medial Gastrocnemius for rows 1–4. All the information is represented across trials and gait velocity (see the legend for more details).

These measurements were normalized to the average $iEMG$ during the *Pre* trials. The relative $iEMG$, also called EMG ratio $\left( {RT_{EMG} } \right)$ (Eq. (4)), was defined to enable the comparison between trials and participants and to determine the effects of the robotic assistance. Figure 7 represents an example of the relative EMG distributions for one patient across trials.

4 $$RT_{EMG} \left( \% \right) = \frac{iEMG}{{\overline{iEMG}_{Pre} }} \cdot 100$$

**Figure 8:**
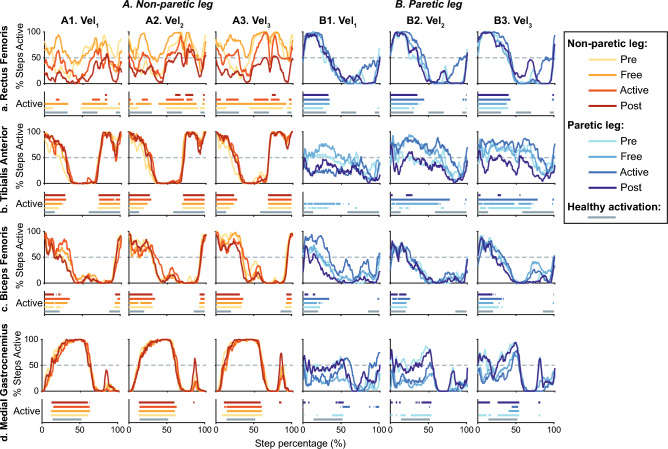
Example of average muscular activation for one patient. If the muscular activity is above the identified threshold, the muscle is considered to be active. Each panel is divided into two subplots; the upper represents the percentage of the total steps in which the muscle is active across a gait cycle, and the lower represents the average activation periods across a gait cycle and compares them with normal healthy timing (grey data). Columns A1–A3 and B1–B3 represent the obtained results during trials performed at the same velocity (namely $vel_{1} ,$ $vel_{2}$ and $vel_{3}$ and for the non-paretic (a's columns) and paretic legs (b’s columns) respectively. Each row shows the activation of a representative muscle: Rectus Femoris, Tibialis Anterior, Biceps Femoris, and Medial Gastrocnemius for rows (**a**–**d**). The information of each leg and trial is represented using a different color and gray lines represent healthy activation^36^ (see the legend for more details).

The timings of EMG signals were evaluated using the Burst Duration Similarity Index ($BDSI$)^29,34^. This metric compares the activation and inhibition periods of two EMG signals. To determine these activity periods, the Teager Kaiser Energy Operator (TKEO) was used to amplify instantaneous energy changes and differentiate between active and relaxed conditions^29,35^. The noise signals acquired during the baseline recordings were used to determine the activation threshold. It was defined as fifteen times the standard deviations over the average baseline signal^35^. Figure 8 represents an example of EMG activation for one patient across trials. If a muscle is active in more than 50% of the steps in a certain step phase, this muscle is considered to be active at this phase.

Given two signals $s_{1}$ and $s_{2}$, of $N$ samples, the BDSI between them requires two binary vectors: $On_{EMG}$ and $Off_{EMG}$, with ‘1’ indicating simultaneous activation or inactivation respectively and ‘0’ otherwise. The BDSI is calculated according to Eq. (5) as described in^29^.

5 $$BDSI\left( \% \right) = f\left( {s_{1} ,s_{2} } \right) = \frac{{sum\left( {On_{EMG} } \right) + sum\left( {Off_{EMG} } \right)}}{N} \cdot 100$$

**Figure 9:**
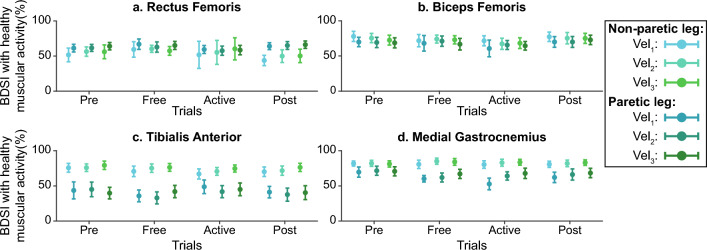
Example of muscular activity timing compared with healthy data for one patient. Data distributions are represented through the mean +/− the standard deviation. Each panel represents information for a representative muscle: Rectus Femoris, Tibialis Anterior, Biceps Femoris, and Medial Gastrocnemius. All the information is represented across trials and gait velocity (see the legend for more details).

Timing analysis compared the muscular activation across trials with healthy activation patterns during normal-velocity gait extracted from^36^. Figure 9 represents an example of the BDSI distributions for one patient across trials.

### Ethical committee

The local ethical committee at Hospital 12 de Octubre gave approval to the experimental protocol (id number: 20/034) and warranted its compliance with the Declaration of Helsinki. Written (signed) informed consent was obtained from all enrolees. Trial and protocol were publicly registered in ClinicalTrials on 17/11/2021; Trial id: NCT05138211.

## Results

### Inter-subject analysis

We aim at evaluating the embodiment of the REFLEX action in the neural gait control of the patients. This embodiment would imply changes in the activity of the lower-limbs muscles that lead to the decrease of the compensation mechanisms developed by the participants and therefore an increase of their gait symmetry. Once the data of each patient was processed, a global analysis was conducted in order to find significant effects in all participants due to the assistance provided by the robot. The metrics’ distributions during a trial were built as follows: the average value during each velocity epoch for each patient was considered, so each patient was represented by four repetitions of the metric.

After checking the heteroscedasticity (Levene test; $P < 0.05$) and non-normality (Kolmogorov–Smirnov test; $P < 0.05$) of some of the data distributions, significant differences between experimental conditions were looked for (Friedman test; $P < 0.05$). The Bonferroni correction was used to counteract the effect of multiple comparisons, and reduce the possibility of Type I errors.

**Figure 10:**
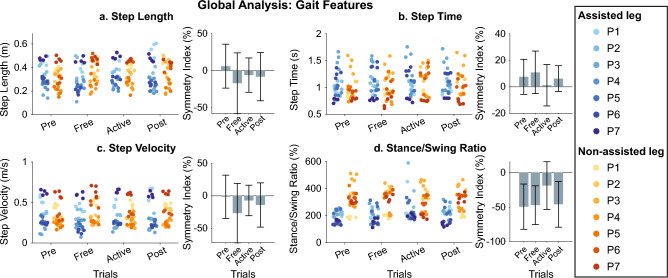
Global inter-subject gait features analysis at $vel_{1}$. Each panel (**a**–**d**) is divided into two subpanels: the left subpanel represents all the metric measurements differentiating trial, leg, and patient, while the right subpanel represents the symmetry index distributions across trials. The bar plot shows the average value + /− the standard deviation; markers represent individual metric values. The same color represents the same patient across the figure (see the legend for details).

**Figure 11:**
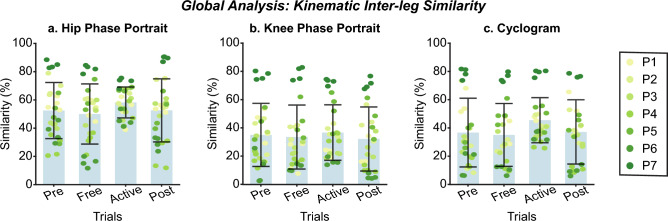
Global inter-subject analysis of interleg kinematic similarity at $vel_{1}$. Each panel (**a**–**c**) represents the interleg kinematic similarity across trials regarding the Hip phase portrait (**a**), the knee phase portrait (**b**), or the hip/knee cyclogram (**c**). The bar plot shows the average value +/− the standard deviation; markers represent individual metric values. The same color represents the same patient across the figure (see the legend for details).

**Figure 12:**
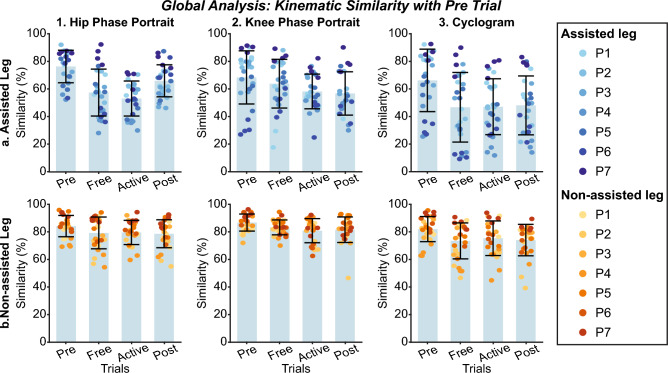
Global inter-subject kinematic intra-leg similarity analysis at $vel_{1}$. Each panel compares the evolution of kinematic representations from *Pre* trials. Columns group the results of hip phase portraits (column 1), the knee phase portraits (column 2), and hip/knee cyclograms (column 3). The first row represents the metric for the assisted leg, while the second row represents the non-assisted leg. The bar plot shows the average value +/− the standard deviation; markers represent individual metric values. The same color represents the same patient across the figure (see the legend for details).

**Figure 13:**
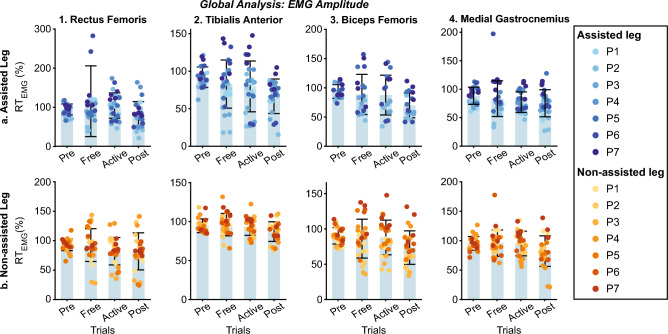
Global inter-subject EMG amplitude analysis at $vel_{1}$. Each panel represents the relative EMG amplitude compared with *Pre* across trials. Columns group the results for the same muscles (Rectus Femoris, Tibialis Anterior, Biceps Femoris, and Medial Gastrocnemius for columns 1–4), and rows group data for the same leg (assisted and non-assisted leg in the first and second row, respectively). The bar plot shows the average value +/− the standard deviation; markers represent individual metric values. The same color represents the same patient across the figure (see the legend for details).

**Figure 14:**
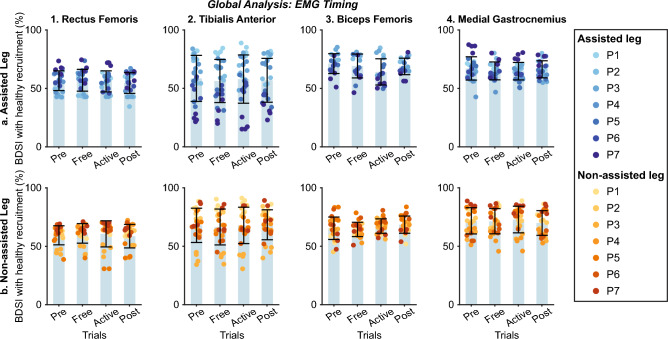
Global inter-subject EMG timing analysis at $vel_{1}$. Each panel represents the BDSI with the normal healthy muscular recruitment across trials. Columns group the results for the same muscles (Rectus Femoris, Tibialis Anterior, Biceps Femoris, and Medial Gastrocnemius for columns 1–4), and rows group data for the same leg (assisted and non-assisted leg in the first and second row, respectively). The bar plot shows the average value +/− the standard deviation; markers represent individual metric values. The same color represents the same patient across the figure (see the legend for details).

From Figs. 10, 11, 12, 13 and 14 represent the distribution of the assessed metrics during $vel_{1}$, including gait features, kinematic analysis and muscular recruitment. However, none of these analyses resulted in significant effects due to robot assistance. Comparisons between different gait speeds did not result in significant differences in the metrics either. Figures S1–S5 of the Supplementary Material show these distributions for $vel_{2}$ and $vel_{3}$.

### Intra-subject analysis

Although this experimental validation did not result in significant global effects due to REFLEX assistance, intra-subject analyses were conducted to evaluate individual changes in any of the evaluated metrics. The objective was to detect individual effects due to the robot’s action, similarly to those obtained in our previous experiment^20^.

The data distributions that characterized each trial and gait velocity were built by pooling together the assessed metrics during each step at the evaluated trial and velocity. The statistical analyses were performed across trials for each gait velocity. Normality (Kolmogorov–Smirnov test; $P>0.05$) and homoscedasticity (Levene test; $P>0.05$) of the distributions involved were assessed. If they were fulfilled, ANOVA tests were used for determining significant differences between distributions ($P<0.001$); otherwise, Kruskal–Wallis tests were used ($P<0.001$). In both cases, the Bonferroni correction was used to counteract the effect of multiple comparisons, and reduce the possibility of Type I errors. Since global results were not found, this analysis was limited to immediate effects; therefore, only *Pre*, *Free* and *Active* trials were compared.

**Figure 15:**
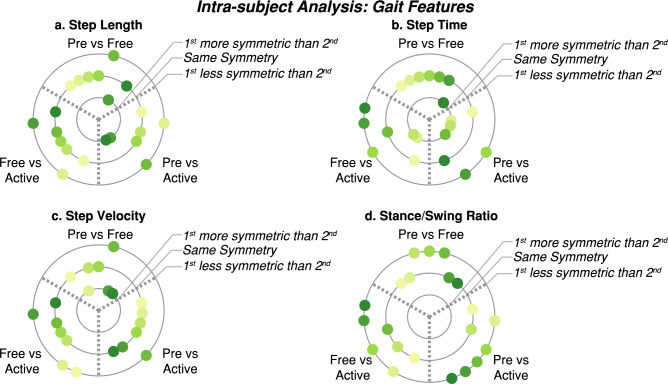
Summary of the intra-subject gait feature analysis at ${\varvec{vel}}_{1}$. Each panel (**a**–**d**) represents the results when comparing the symmetry of a gait feature between *Pre*, *Free* and *Active* trials. Each subject is represented with a different color and the same color represents the same patient across panels.

**Figure 16:**
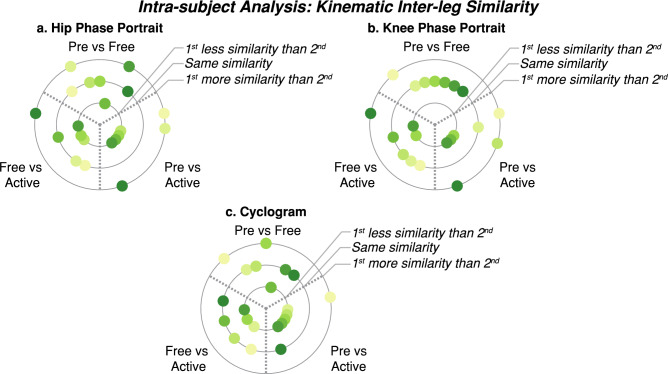
Summary of the intra-subject kinematic inter-leg similarity analysis at ${\varvec{vel}}_{1}$ Each panel (**a**–**c**) represents the results when comparing the similarity between limbs of different kinematic representations between *Pre*, *Free* and *Active* trials. Each subject is represented with a different color and the same color represents the same patient across panels.

Figures 15 and 16 summarize the individual results of this gait feature and inter-leg similarity analysis for each patient during $ve{l}_{1}$ as examples. Figures S6–S8 of the Supplementary Material show the rest of the analysis at $vel_{1}$ and Figs. S9–S13 of the Supplementary Material show these individual results for $vel_{2}$ and $vel_{3}$. As it can be seen, although there are significant differences between trials within a patient. None of the patients responded in the same way and even some of them presented contrary behaviors, showing an increase or a decrease of the same metric under the same conditions. For instance, step length symmetry or step time symmetry responded differently to robot assistance, as some patients increased these symmetries while others decreased them. Another example of different participants’ behavior is the muscular response to assistance, independently of the muscle or leg assessed.

## Discussion

This study was conducted to evaluate the embodiment of the REFLEX's actions by stroke survivors during assisted gait. During our pilot experiment with three stroke survivors^20^, results seemed to indicate that REFLEX’s assistance could improve the gait symmetry of hemiparetic subjects or, at least, compensate for the disturbance introduced by the own device, although we did not evaluate if the origin of this improvement were adaptations in the neural gait control that should be manifested in variations of the muscular activity.

In this study, we aimed at addressing the mentioned limitation of our previous work, and also we increased the sample size of the stroke survivors involved in the experiment. However, our results indicate that the effect of REFLEX was heterogeneous across patients since some of them improved their gait symmetry according to the gait metrics and the kinematic measurements, while others reported a less symmetric gait, reducing step length or step time symmetry or the inter-leg similarity of hip and knee phase portraits for instance.

The effect of the robot assistance in the paretic knee movement was not as evident as during the pilot experiment^20^. We believe this difference is because the exoskeleton was better tailored for the first group of participants than for the second one. The prototype adjustment is limited, and a worse joint alignment, which results in worse force transmission, may be responsible for this poor exoskeleton's performance. In this sense, the correct alignment of joint and motor axis should be ensured to solve this problem. During the experiment, we also observed that the anthropometry of the patient had a relevant effect in the force transmission between the device and the patient's body, especially in patients with excess of soft tissues in which the exerted force is dedicated to deform them. In this sense, it is necessary to include a better tailoring system in the prototype to be adjustable for a wider range of patients. According to Babic et al.^37^, this is a common problem in the field as joint alignment and efficient power transmission are key factors for the proper function of these devices.

The main outcomes obtained from this experiment were the high variance and heterogeneous behaviors of all the evaluated metrics, which emphasizes the singularities of each patient to cope with the provided assistance. This high dispersion is present not only in the metrics related to the muscular activity, as previously reported in^26^, but also in the kinematic and gait metric results. The main consequence of this variability was that no global effects were identified due to REFLEX assistance. Although the metrics showed a positive evolution due to assistance during the preliminary validation, this behavior could not be extrapolated to the group.

Despite significant group effects not being detected, it does not mean that REFLEX assistance had no effects on the patients. Actually, significant effects were identified at the subject level but were not consistent among them. The high variance in the attained results is responsible for not being able to accept neither reject the initial hypothesis about the embodiment of the robotic device, since a larger sample size would have been necessary. All the evaluated metrics showed significant positive effects in one subject at least; however, contrary effects were also reported. These different patient reactions under the same assistance could be due to the heterogeneity of the patients, since the time after the stroke varies from eight to 49 months, which also implies that they are in different phases of the rehabilitation process, or the FIM score varies from 79 to 126 and the Time and Go test from 7.46 to 37.62 s, which implies that the disability degree of the patient is also heterogeneous. In addition, the stroke may affect to different brain regions, which also implies differences in the abilities of each patient. These different characteristics and responses reinforce the necessity of properly tailoring the robot to each patient individually to adapt the exoskeleton to each subject and therefore maximize its performance^26^. In this sense, customizing the action of the robot to the concrete patient needs may boost the benefits of the provided assistance. This customizing process may include variable assistance levels that would be adapted to the ability of the patient in each gait sub-phase, or a programmable advance/delay in the action of the robot that would make converge its action with the user pre-estimation.

A plausible explanation for this high variance in the measured outcomes might be the varying conditions of the experiment. The experimental protocol was based on the results of Haufe et al.^38^, who reported kinematic and muscular adaptation after one minute of using a knee exoskeleton. However, they recruited healthy subjects instead of stroke survivors. Stroke patients may require longer adaptation periods to incorporate the robot's actions. We believe that longer trials without velocity changes would imply more stable experimental conditions that would favor reaching the gait’s steady-state and, therefore, potentially reduce the variance of the measured outcomes. This improvement in the experimental procedure would also facilitate the embodiment of the robot assistance by the patient, since repetitiveness improves matching the patient’s expectation and robot's action. These aspects will be addressed in future experiments.

Muscular reactions were not homogeneous across patients either. Some of them reduced muscular activation amplitude while others increased it or did not show any variation, independently of whether the assessed leg was the assisted or the unassisted one. Similarly, recruitment timing responses were heterogeneous, remaining unchanged, increasing or decreasing the timing similitude with the healthy activation indistinctly.

Our initial hypothesis was that the assistance provided by REFLEX would lead to variations in muscular activity and compensatory strategies. However, although some significant variations were identified for individual participants, no global effects were found. This contrasts with the results of previous experiments, where the assistance provided by a robotic exoskeleton reduced the activity of the involved muscles^13,21–25^. Schmalz et al.^39^ reported a reduction in the compensatory strategies developed by hemiparetic subjects due to the assistance of a knee robotic exoskeleton. However, none of the six patients involved in the study had suffered a stroke, but their hemiparesis was due to different underlying conditions. Another previous study involved stroke survivors and reported muscular changes^28^, although the authors carried out a complete rehabilitation therapy and the reported muscular variations were after it. Therefore these changes are not only due to the assistance but also due to the therapeutic effect of the intervention.

Androwis et al.^29^ also reported muscular adaptation in stroke patients during a unique simulated therapy session. Their results showed a significant increase in muscular activity in the Rectus Femoris and Soleus of the paretic leg and better activation timing for the affected Vastus Lateralis and Rectus Femoris. Although muscular reactions were reported in this case, two main differences are present compared with the presented approach. On the one hand, they used the EksoGT prototype, a complete lower-limb robotic exoskeleton for gait training, instead of a single-joint knee exoskeleton. On the other hand, their purpose was gait rehabilitation instead of gait assistance. In this regard, they looked for a higher muscle involvement in the paretic leg instead of reducing compensatory strategies and muscular activity in the nonparetic leg.

Although some of the participants involved in this REFLEX validation showed significant effects in their muscular recruitment, these were not correlated with kinematics modifications. This fact indicates that the kinematic variations are more likely due to the effects of the extra load and the forces exerted by REFLEX than to the integration of the REFLEX’s actions by the Nervous System. If these actions had been properly embodied, the nervous system would have modified the muscular activity in consequence, but this adaptation did not occur.

When comparing the reported results in the published literature and those obtained in this experiment, although the conclusions seem to be opposed, there is an important difference that it is necessary to highlight. The above-mentioned articles that reported a reduction in the compensation mechanisms or the proper integration of the assistance while walking did not involve stroke patients but healthy or hemiparetic walking subjects with a different underlying condition.

Stroke may directly affect the integration of afferent signals by the central nervous system, leading to proprioception deficits in several cases^40,41^. Actually, the motor-learning ability of a subject depends on their central proprioceptive processing^42^. This proprioceptive impairment due to stroke may also be a reason for the different effects between healthy and stroke populations when assisted by a robotic exoskeleton. If the central nervous system is not able to properly process the afferent information that reports the device's action, it is not possible for the central nervous system to react appropriately in consequence. However, if this integration occurs exclusively at the spinal level, which remained unaffected due to stroke, our hypothesis would be valid, and the robot’s assistance would lead to the reduction of compensatory mechanisms. Research efforts are needed in this regard, so the ability of stroke survivors to detect and process the assistive action of the robot would be fully understood.

Other future work line opened is to assess the embodiment of robotic exoskeletons in stroke patients to understand its underlaying mechanisms. Extra analysis can be carried out following the same methodology previously reported to assess the embodiment of prosthesis or wheelchairs^43^. In this regard, subjective questionnaires or personal interviews could be used to evaluate if users consider the device as a part of their own body^44,45^ or the proprioceptive drift could be measured as a direct and objective sign of a change in the body schema^46,47^.

Some limitations have been identified in this study. The heterogeneity in the patients involved in the experiments and the varying experimental conditions can be responsible of the high variability of the experimental data. In addition, the limited adjustability of the prototype may prevent obtaining the optimum result for each patient. We acknowledge that these limitations may hamper the generalizability of the results obtained in the current experimentation. However, we have detected several aspects that should be considered when analyzing the embodiment of robotic exoskeletons by stroke patients, such as the consequences of varying experimental conditions, heterogeneity of subjects involved and implication of hampered processing of afferent information due to stroke.

## Conclusions

This paper has presented an experimental validation carried out with the REFLEX prototype involving stroke survivors to evaluate the embodiment of this device. The aim of this validation was to further understand how stroke survivors integrate the robot's actions. However, the high variability of the obtained results avoided confirming or rejecting the original hypothesis.

Although global assistance effects were not identified, significant effects were reported at the subject level with each subject responding differently to the assistance. Notably, differences were found between how the assistance affected the stroke patients that participated in this study and how healthy subjects responded to robotic assistance, according to other studies reported in the literature. More research would be needed to fully understand the implications of the stroke's consequences in how patients acquire the action of robotic exoskeletons and if they are able to embody it properly.

### Supplementary Information

Supplementary Information.

## Data Availability

The datasets used and/or analysed during the current study are available from the corresponding author on reasonable request.
