# Supplementary material for: Robotic exoskeleton embodiment in post-stroke hemiparetic patients: an experimental study about the integration of the assistance provided by the REFLEX knee exoskeleton
Source: Sci Rep. 2023 Dec 21;13:22908. doi: 10.1038/s41598-023-50387-8 (PMC10739721; doi:10.1038/s41598-023-50387-8)
Supplement: Supplementary file 1 — Supplementary Information. [file 41598_2023_50387_MOESM1_ESM.pdf]

Robotic exoskeleton embodiment in post-stroke hemiparetic patients: an experimental study about the integration of the assistance provided by the REFLEX knee exoskeleton

Supplementary Material.

Julio Salvador Lora-Millán[1]; Francisco José Sanchez-Cuesta[,2,3]; Juan Pablo Romero[2,3,4]; Juan C. Moreno[5]; Eduardo Rocon[6]

1. Electronic Tecnology Area, Rey Juan Carlos University, Madrid, Spain
2. Facultad de Ciencias Experimentales, Universidad Francisco de Vitoria, Pozuelo de Alarcón, Madrid, Spain
3. Brain Injury and Movement Disorders Neurorehabilitation Group (GINDAT), Institute of Life Sciences, Francisco de Vitoria University, Pozuelo de Alarcón, Spain
4. Brain Damage Unit, Hospital Beata María Ana, Madrid, Spain
5. Neural Rehabilitation Group, Cajal Institute, Spanish National Research Council (CSIC), Madrid, Spain
6. Centro de Automática y Robótica, Spanish National Research Council (CSIC), Madrid, Spain

* Corresponding author: julio.lora@urjc.es

Supplementary Table 1. Stroke subject's demographic data

Id Subject	***P1***	***P2***	***P3***	***P4***	***P5***	***P6***	***P7***	***Average*** [a]
Age	57	63	63	68	56	56	57	60±4.69
Heigth (m)	1.7	1.8	1.7	1.7	1.7	1.7	1.7	1.74±0.05
Weight (kg)	84	84	83	74	81	76	76	79.71±4.27
Time after stroke (months)	9	26	38	21	49	43	8	27.71±16.22
MMSB Score	29	27	13	26	26	25	29	25±5.51
FIM Score	117	104	79	118	90	107	126	105.86±16.61
Time Up and Go (s)	24.66	28.07	37.52	20.86	29.62	18.35	7.46	23.79±9.54
Sex	Male	Male	Male	Female	Male	Female	Male	
Stroke	Subcortical ischemic	Cortical ischemic	Cortical ischemic	Cortical hemorrhagic	Cortical hemorrhagic	Cortical hemorrhagic	Cortical hemorrhagic	
Paretic side	Right	Left	Right	Right	Left	Right	Left	

[a] Mean ± STD

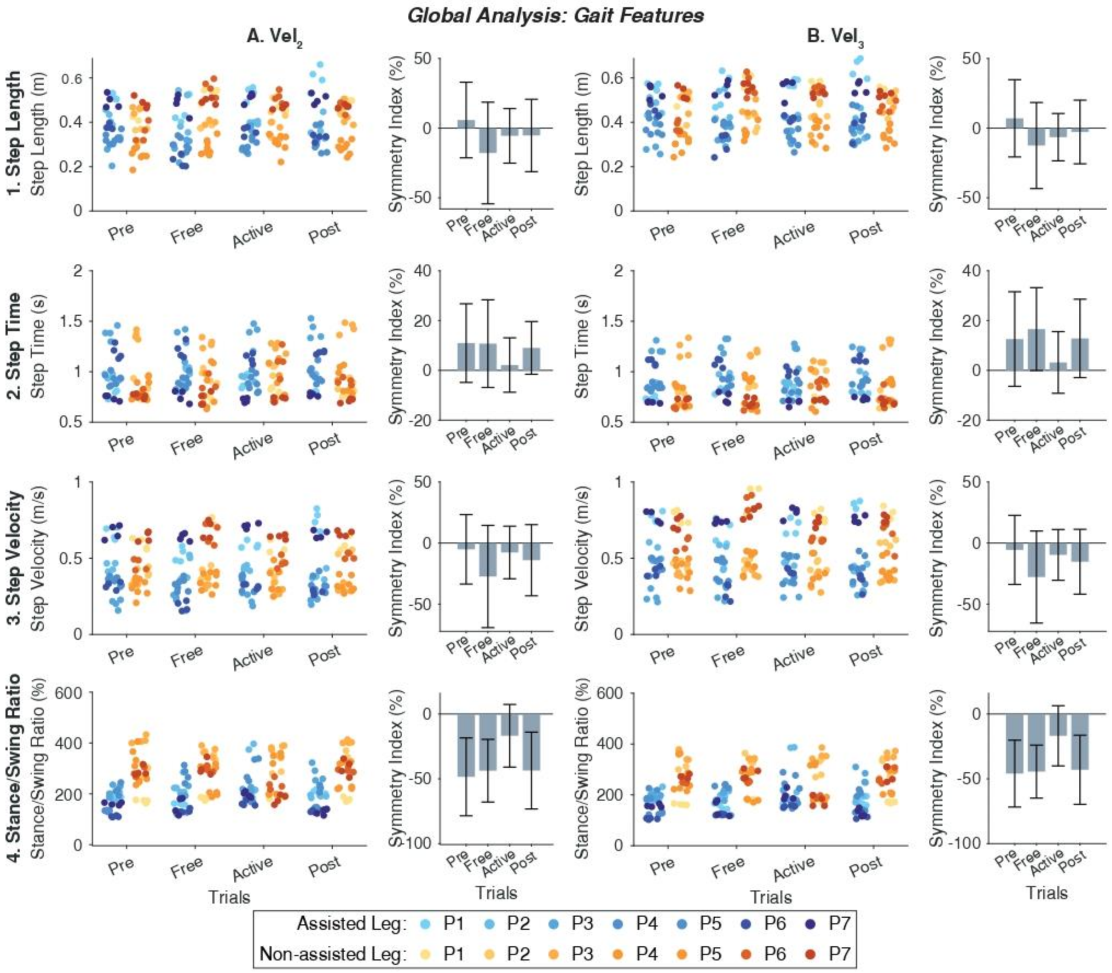

Supplementary Figure S1. Global inter-subject gait features analysis at vel_2 (column A) and vel_3 (column B). Each row (1-4) represents a different metric. Each cell is divided into two subpanels: the left subpanel represents all the metric measurements differentiating between trial, leg, and patient, while the right subpanel represents the symmetry index distributions across trials. The bar plot shows the average value +/- the standard deviation; markers represent individual metric values. The same color represents the same patient across the figure (see the legend for details).

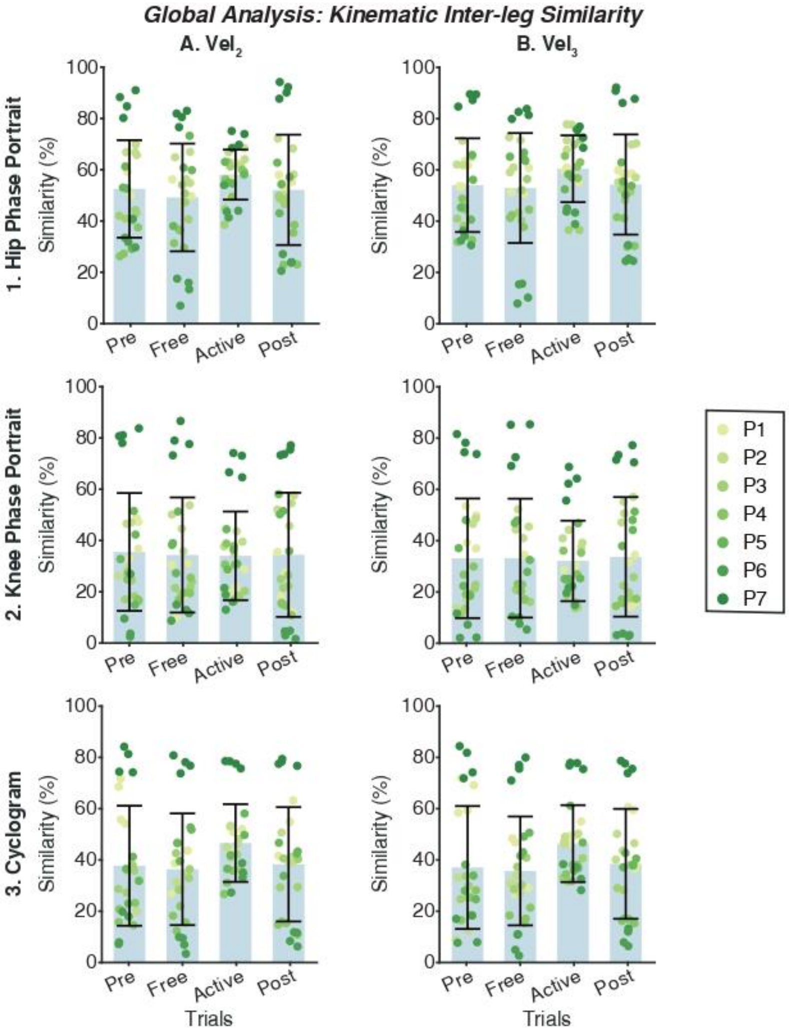

Supplementary Figure S2. Global inter-subject analysis of interleg kinematic similarity at vel_2 (column A) and vel_3 (column B). Each row (1-3) represents the interleg kinematic similarity across trials regarding the hip phase-portrait (1), the knee phase portrait (2), or the hip/knee cyclogram (3). The bar plot shows the average value +/- the standard deviation; markers represent individual metric values. The same color represents the same patient across the figure (see the legend for details.

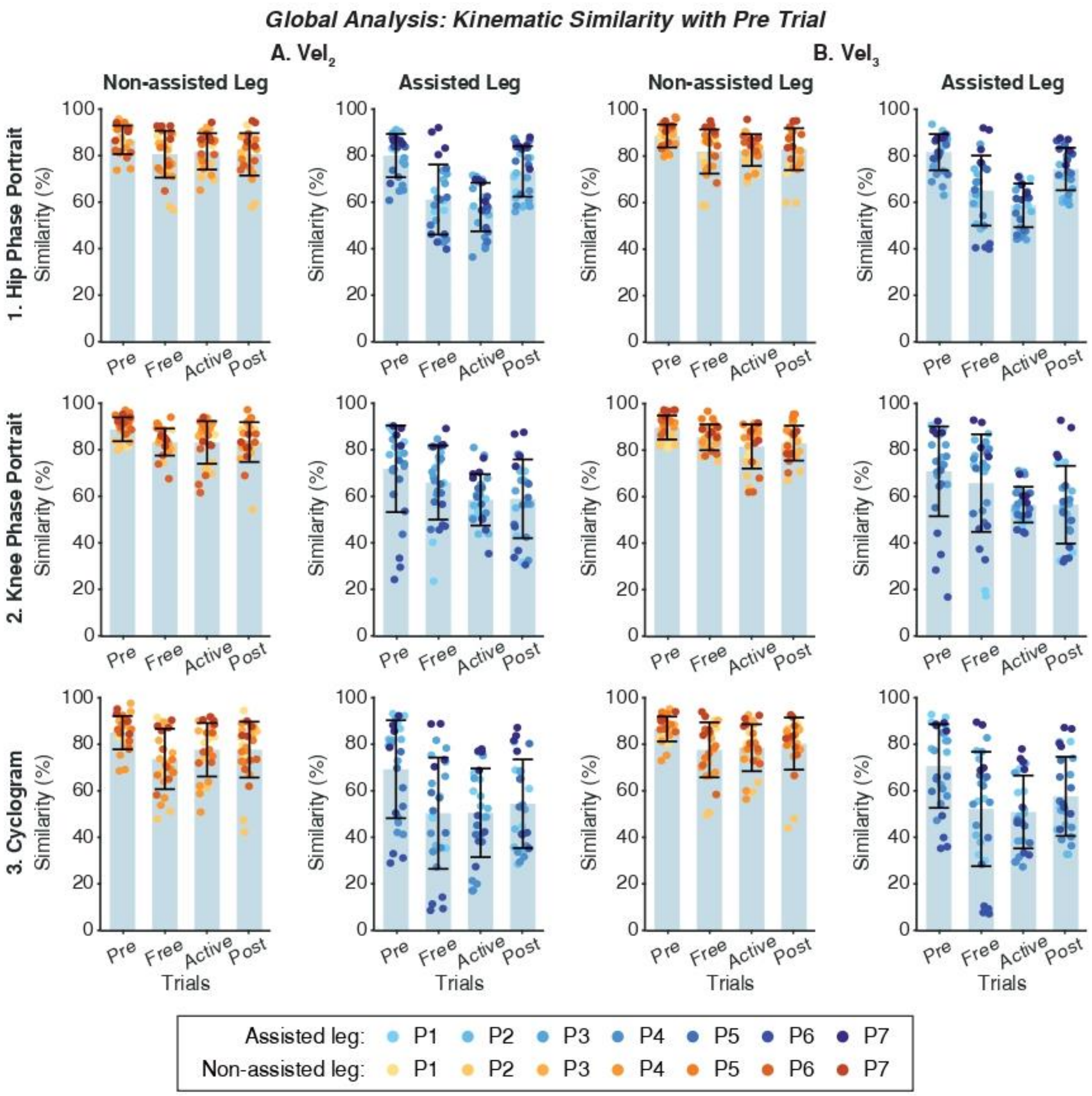

Supplementary Figure S3. Global inter-subject kinematic intra-leg similarity analysis at *vel₂* (column A) and *vel₃* (column B). Each graph compares the evolution of kinematic representations from \textit{Pre} across trials. Rows group the results of hip phase portraits (row 1), the knee phase portraits (row 2), and hip/knee cyclograms (row 3). The first columns represent the metric for the non-assisted leg, while the second columns represent the assisted leg. The bar plot shows the average value +/- the standard deviation; markers represent individual metric values. The same color represents the same patient across the figure (see the legend for details).

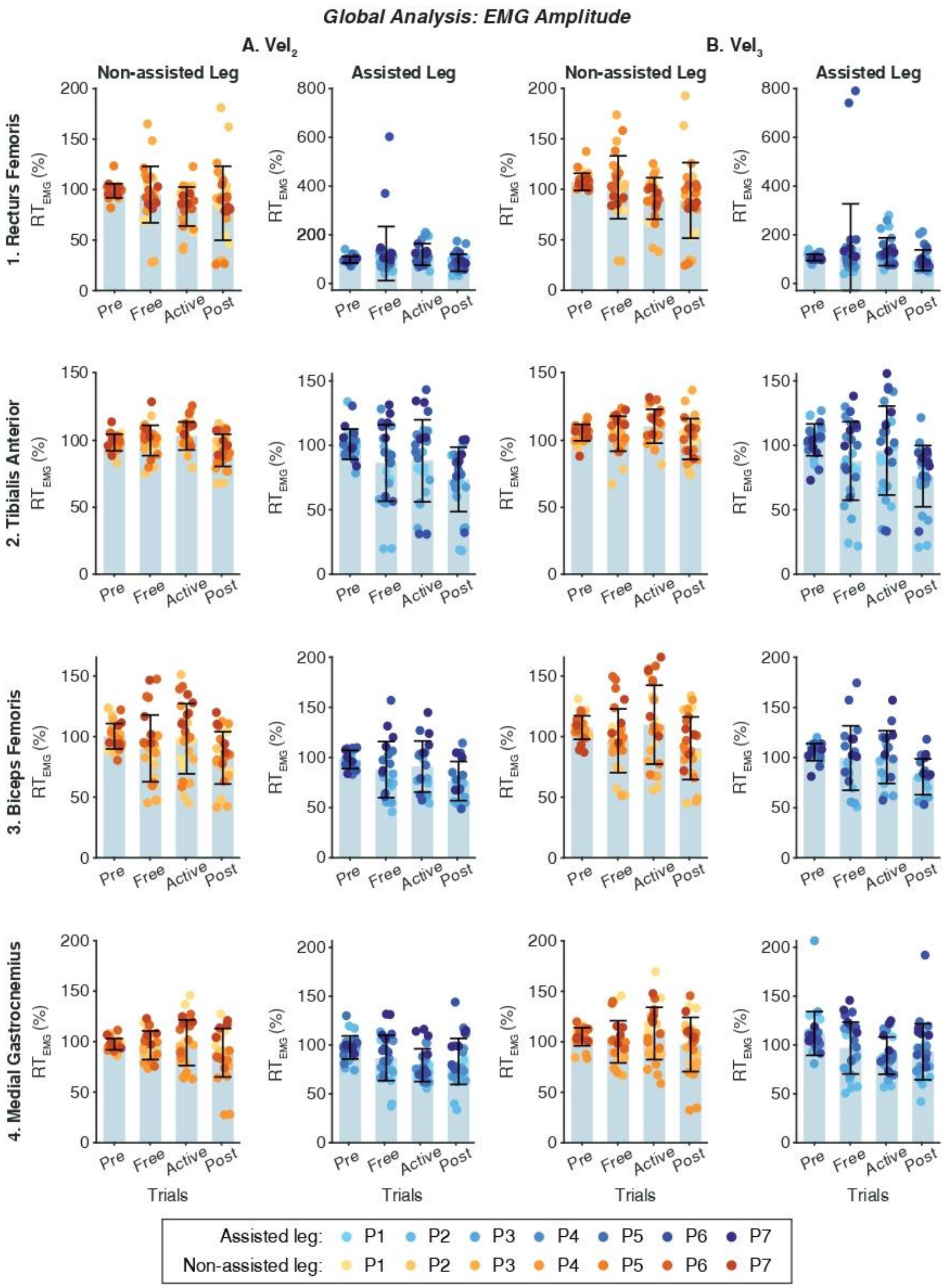

Supplementary Figure S4. Global inter-subject EMG amplitude analysis at vel_2 (column A) and vel_3 (column B). Each represents the EMG ratio across trials. Rows group the results for each muscle (Rectus Femoris, Tibialis Anterior, Biceps Femoris, and Medial Gastrocnemius for rows 1-4, respectively). The first columns represent the metric for the non-assisted leg, while the second columns represent the assisted leg. The bar plot shows the average value +/- the standard deviation; markers represent individual metric values. The same color represents the same patient across the figure (see the legend for details).

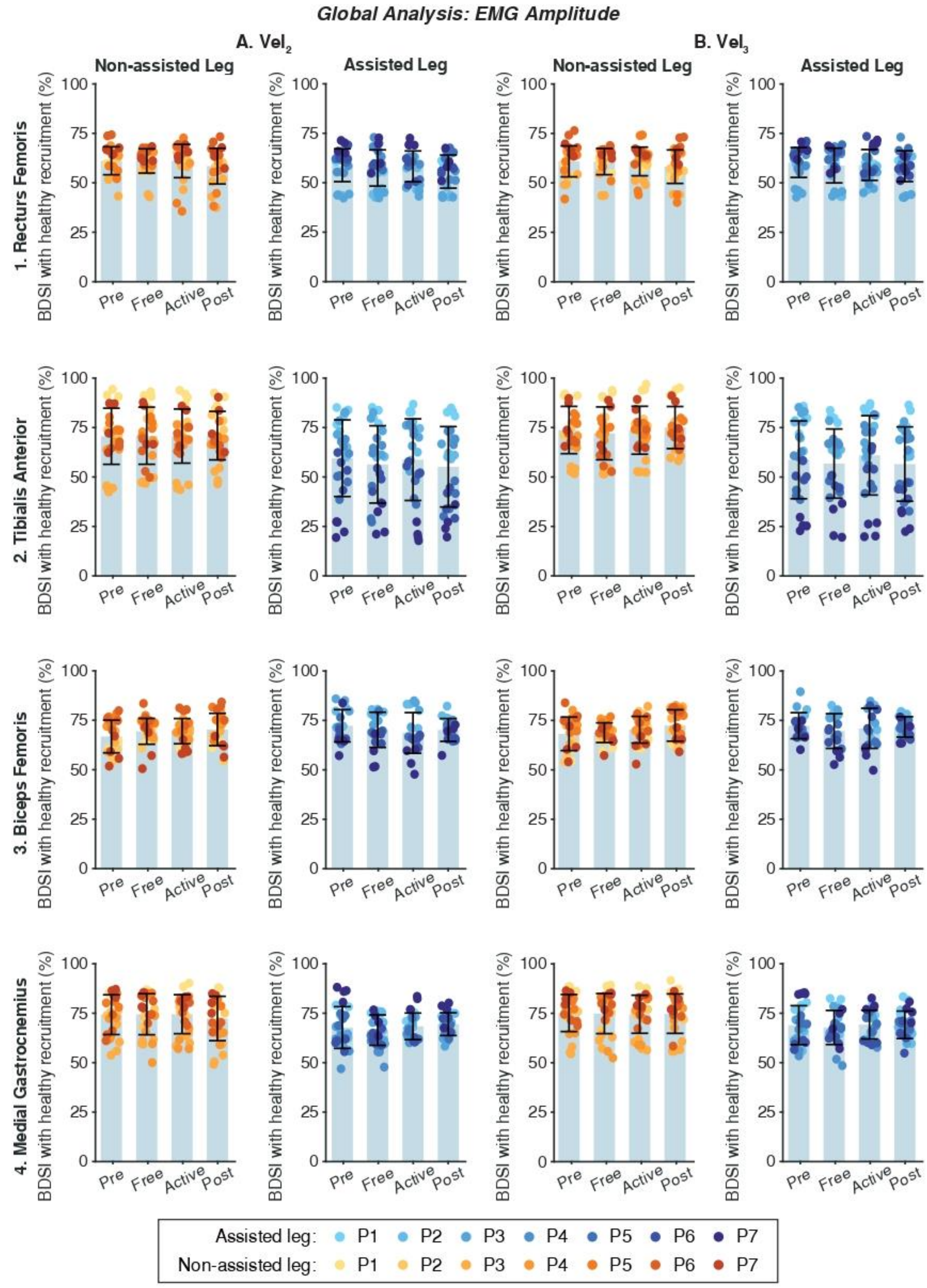

Supplementary Figure S5. Global inter-subject EMG timing analysis at *vel*₂ (column A) and *vel*₃ (column B). Each represents the BDSI with the normal healthy muscular recruitment across trials. Rows group the results for each muscle (Rectus Femoris, Tibialis Anterior, Biceps Femoris, and Medial Gastrocnemius for rows 1-4, respectively). The first columns represent the metric for the non-assisted leg, while the second columns represent the assisted leg. The bar plot shows the average value +/- the standard deviation; markers represent individual metric values. The same color represents the same patient across the figure (see the legend for details).

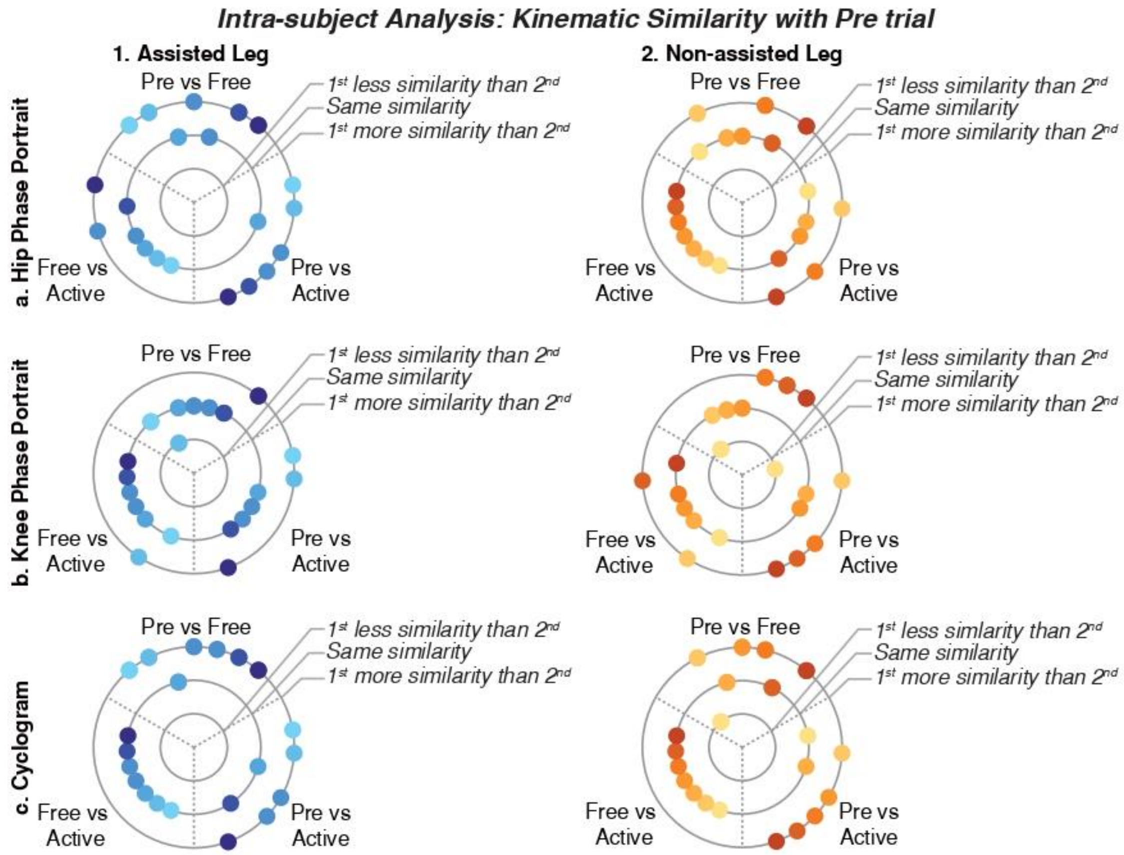

Supplementary Figure S6. Summary of the intra-subject kinematic intra-leg similarity analysis at vel_1. Each panel represents the evolution of kinematic representations from \textit{Pre}, they also compare this evolution in *Pre*, *Free* and *Active* trials. Columns group the results for the same leg (assisted leg in the column 1, and non-assisted leg in column 2). Rows group results for the same kinematic representations (hip phase portrait, knee phase portrait and cyclogram for rows a-c). Each subject is represented with a different color, the same brightness gradation represents the same patient across panels.

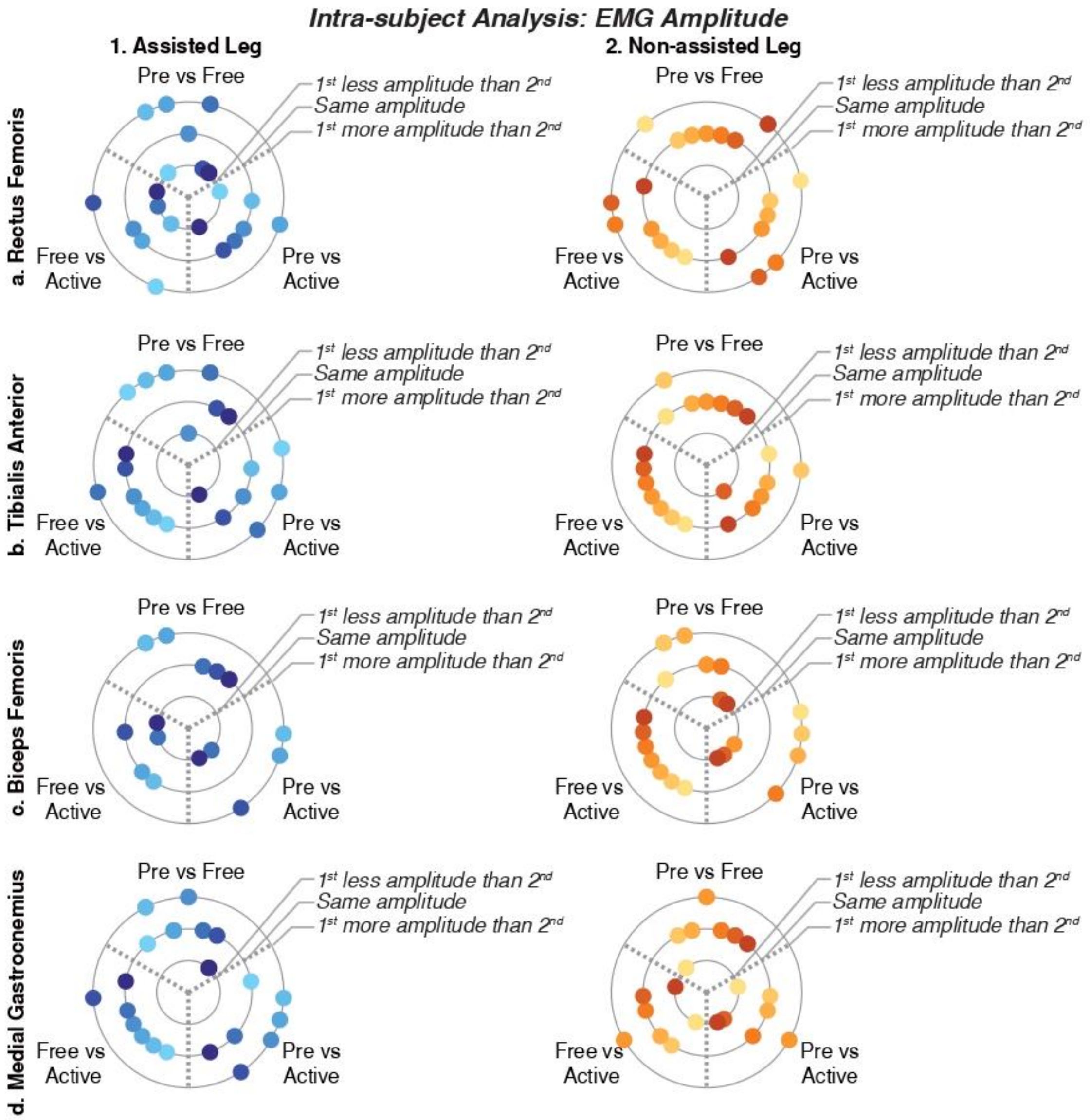

Supplementary Figure S7. Summary of the intra-subject EMG amplitude analysis at *vel*$_1$. Each panel compares the EMG amplitude for different muscles between *Pre*, *Free* and *Active* trials. Columns group the results for the same leg (assisted leg in the column 1, and non-assisted leg in column 2). Rows group results for the same muscles (Rectus Femoris, Tibialis Anterior, Biceps Femoris and Medial Gastrocnemius for rows a-d). Each subject is represented with a different color, the same brightness gradation represents the same patient across panels.

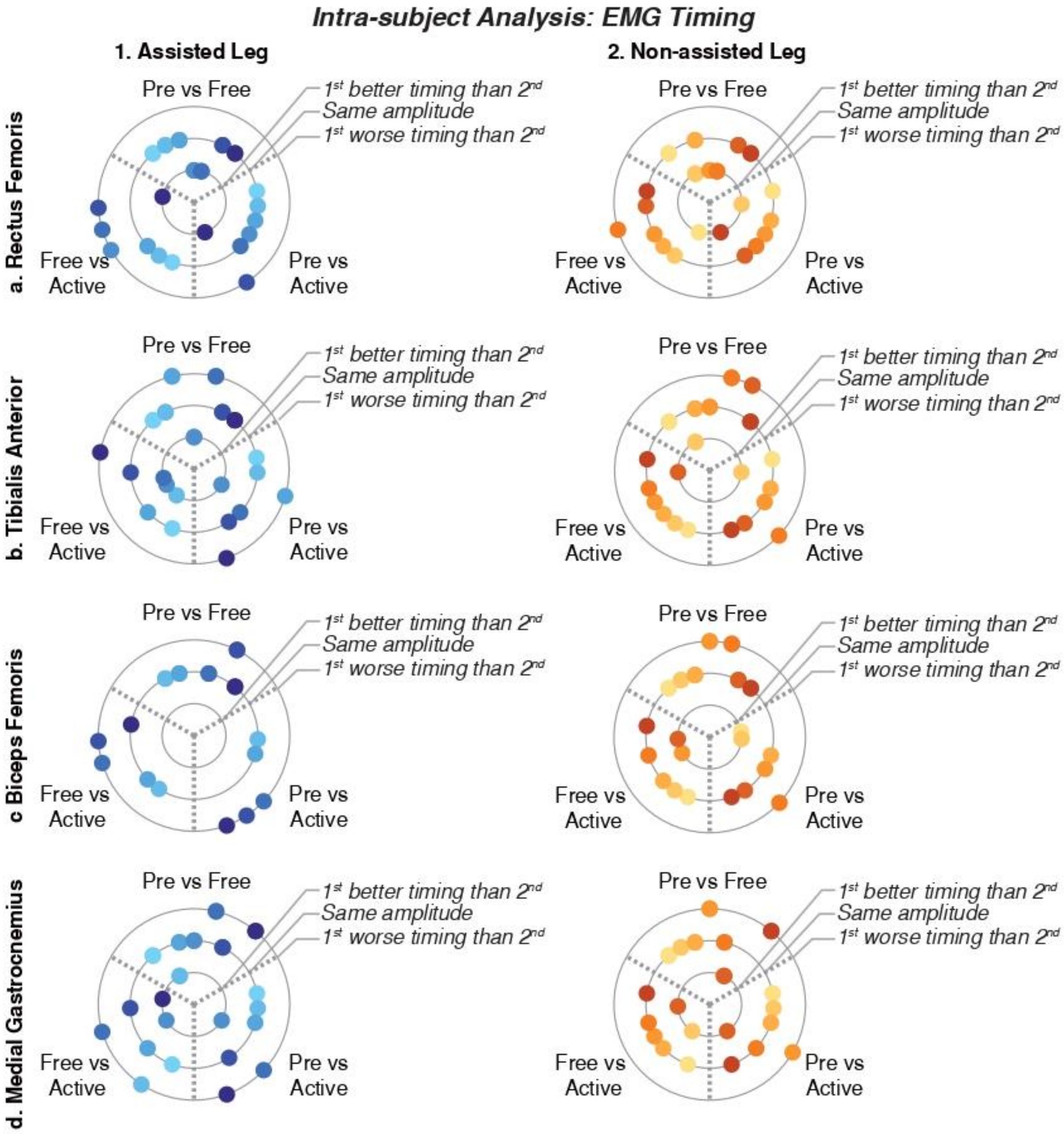

Supplementary Figure S9. Summary of the intra-subject EMG timing analysis at vel_1. Each panel compare the BDSI calculated respect to healthy activation for different muscles between *Pre*, *Free* and *Active* trials Columns group the results for the same leg (assisted leg in column 1 and non-assisted leg in column 2). Rows group results for the same muscles (Rectus Femoris, Tibialis Anterior, Biceps Femoris, and Medial Gastrocnemius for rows a-d). Each subject is represented with a different color, the same brightness gradation the same patient across panels

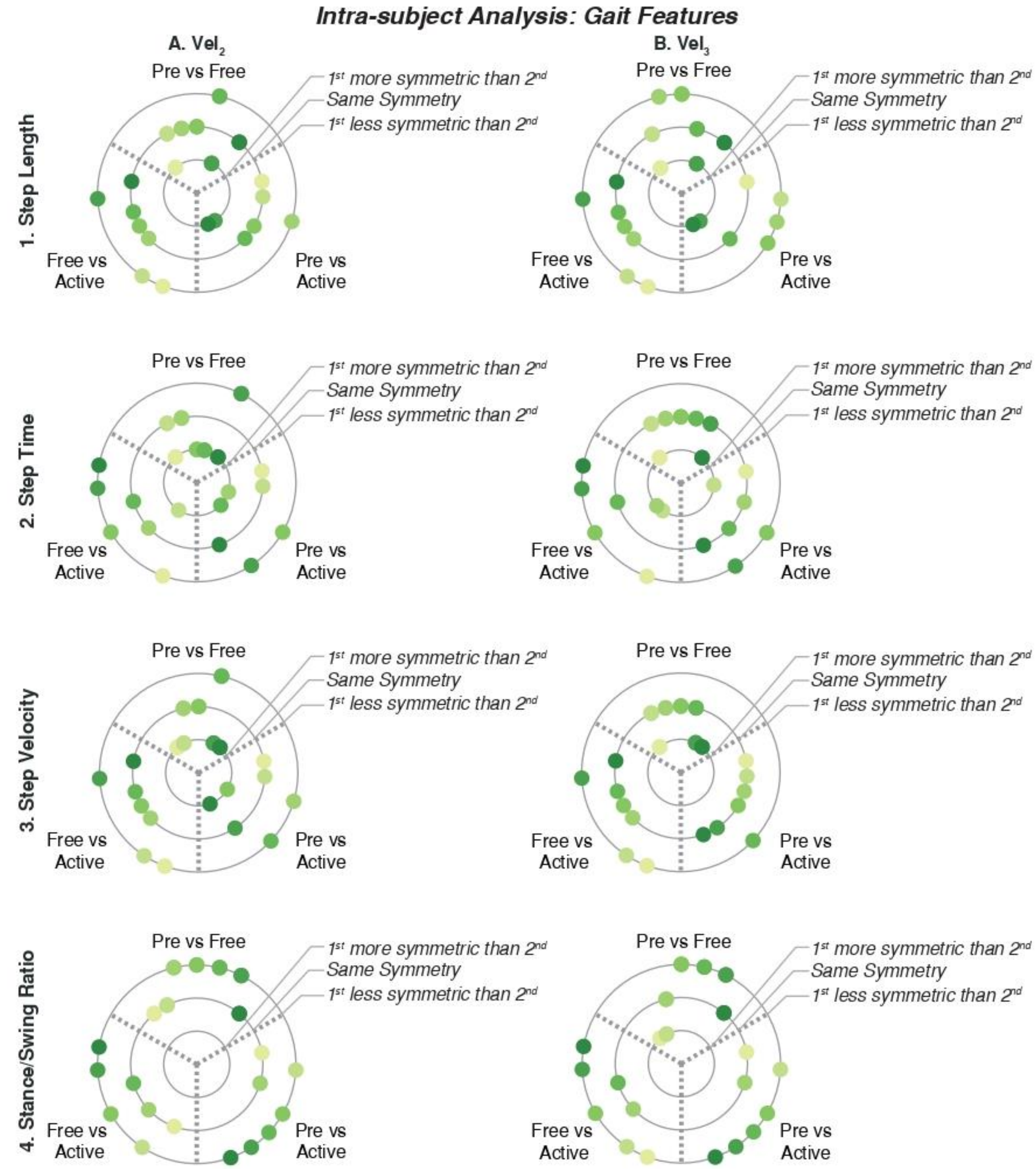

Supplementary Figure S10. Summary of the intra-subject gait feature analysis at *vel*$_2$ (column A) and *vel*$_3$ (column B). Each row (1-4) represents the results when comparing the symmetry of a gait feature between *Pre*, *Free* and *Active* trials. Each subject is represented with a different color and the same color represents the same patient across panels.

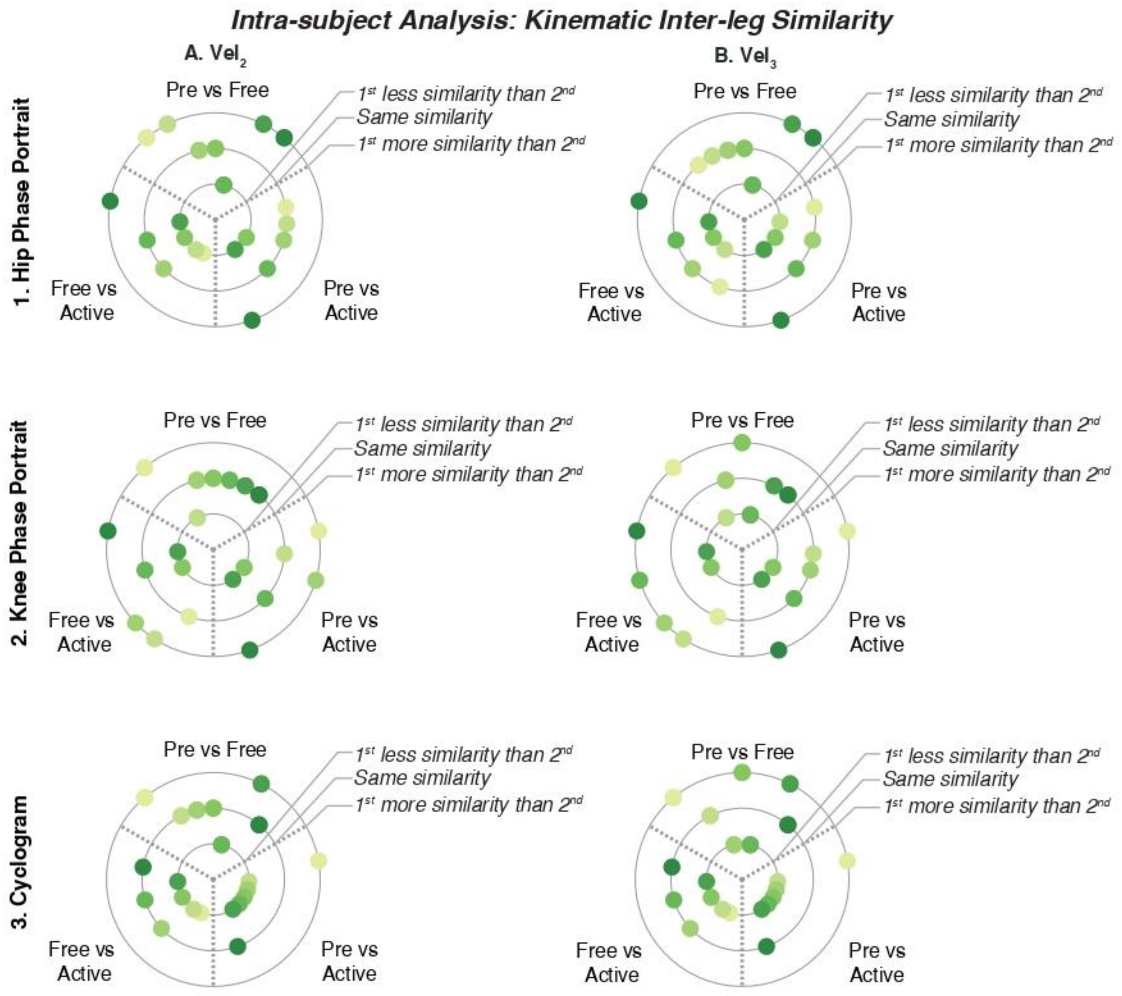

Supplementary Figure S11. Summary of the intra-subject kinematic inter-leg similarity analysis at vel_2 (column A) and vel_3 (column B). Each row (1-3) represents the results when comparing the similarity between limbs of different kinematic representations between *Pre*, *Free* and *Active* trials. Each subject is represented with a different color and the same color represents the same patient across panels.

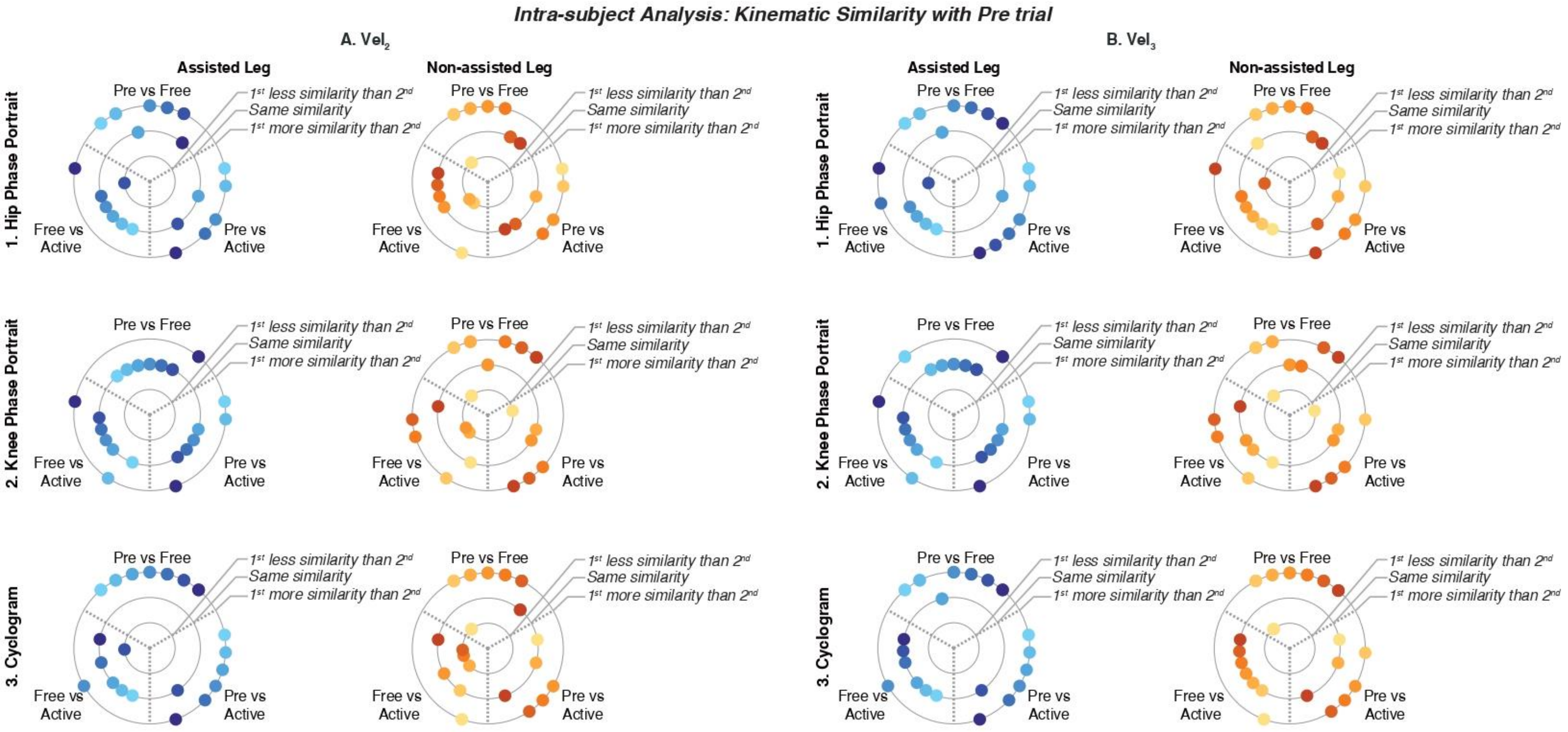

Supplementary Figure S12. Summary of the intra-subject kinematic intra-leg similarity analysis at *vel*$_2$ (column A) and *vel*$_3$ (column B). Each row (1-3) represents the results when comparing the similarity between limbs of different kinematic representations *Pre*, *Free* and *Active* trials. Each subject is represented with a different color, the same brightness gradation represents the same patient across panels.

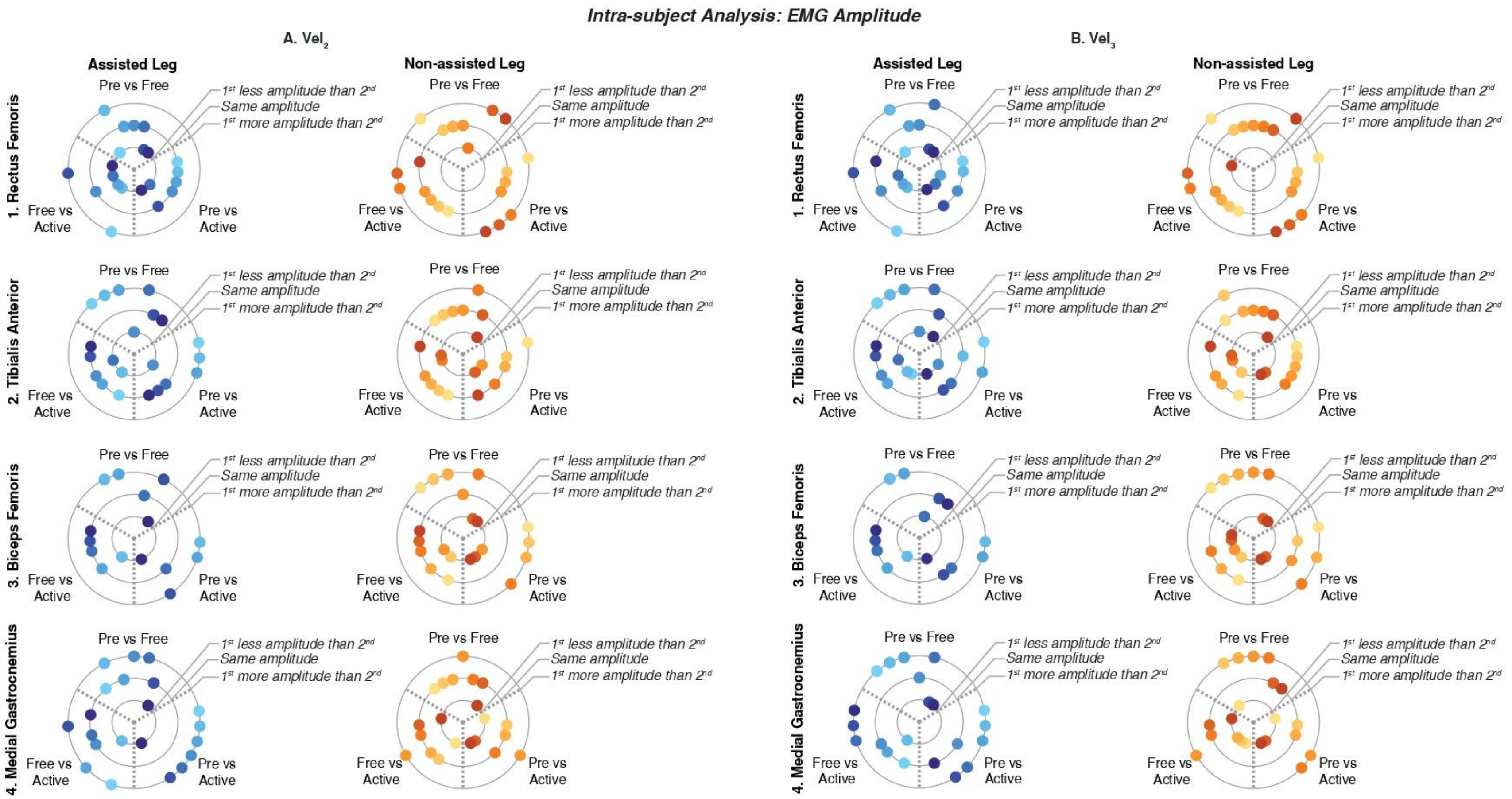

Supplementary Figure S13. Summary of the intra-subject EMG amplitude analysis at *vel*$_2$ (column A) and *vel*$_3$ (column B). Each graph compares the EMG amplitude for different muscles between *Pre*, *Free* and *Active* trials. Columns group the results for the same leg (assisted leg in the first column, and non-assisted leg in the second column of each panel). Rows group results for the same muscles (Rectus Femoris, Tibialis Anterior, Biceps Femoris and Medial Gastrocnemius for rows 1-4).Each subject is represented with a different color, the same brightness gradation represent the same patient across panels.

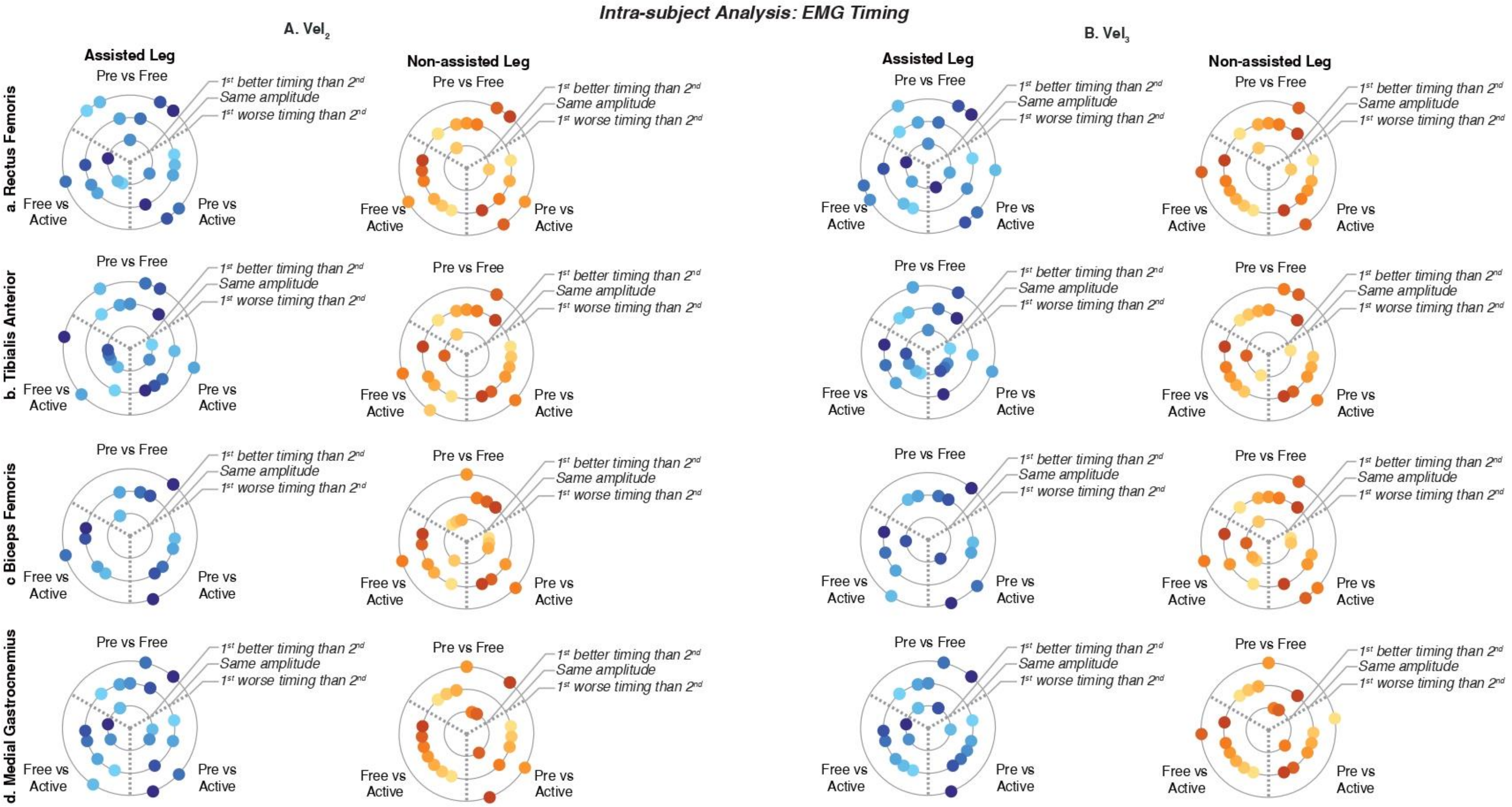

Supplementary Figure S14. Summary of the intra-subject EMG timing analysis at *vel*$_2$ (column A) and *vel*$_3$ (column B). Each panel compare the BDSI calculated respect to healthy activation for different muscles between *Pre*, *Free* and *Active* trials. Columns group the results for the same leg (assisted leg in the first column, and non-assisted leg in the second column of each panel). Rows group results for the same muscles (Rectus Femoris, Tibialis Anterior, Biceps Femoris and Medial Gastrocnemius for rows 1-4).Each subject is represented with a different color, the same brightness gradation represents the same patient across panels.
